# Efficacy of Hwangryunhaedok-tang (Huang-lian-jie-du-tang, Oren-gedoku-to) for patients with hyperlipidemia: a study protocol for a randomized, double-blind, placebo-controlled, parallel, investigator-initiated clinical trial

**DOI:** 10.1186/s13063-020-04695-3

**Published:** 2020-08-27

**Authors:** Boram Lee, Kyungsun Han, Hyo-Ju Park, Ae-Ran Kim, O-Jin Kwon, Changsop Yang, Chung-Sik Cho

**Affiliations:** 1 Clinical Medicine Division, Korea Institute of Oriental Medicine, Yuseong-daero 1672, Yuseong-gu, Daejeon, 34054 Republic of Korea; 2 Department of Internal Medicine, Daejeon Korean Medicine Hospital of Daejeon University, Daedeok-daero 176 beon-gil 75, Seo-gu, Daejeon, 35235 Republic of Korea

**Keywords:** Herbal medicine, Hwangryunhaedok-tang (Huang-lian-jie-du-tang, Oren-gedoku-to), Hyperlipidemia, Korean traditional medicine, Randomized controlled trial

## Abstract

**Background:**

The prevalence of hyperlipidemia continues to increase due to aging and lifestyle changes. Statins are currently used as the first choice for treating hyperlipidemia, but are limited by adverse reactions. Hwangryunhaedok-tang (HHT) has received attention as a promising intervention for hyperlipidemia through a few experimental and clinical trials. This study aims to explore the feasibility, effectiveness, and safety of HHT for hyperlipidemia treatment.

**Methods:**

This is a study protocol for a randomized, double-blind, placebo-controlled, parallel, investigator-initiated, pilot clinical trial held in Daejeon, Republic of Korea. Thirty patients with hyperlipidemia will be randomly allocated to HHT or placebo granule groups in equal proportions. Participants will be administered HHT or placebo granules three times per day for 8 weeks and followed up for another 4 weeks. The primary outcome is low-density lipoprotein cholesterol at 8 weeks from the commencement of treatment. Other blood lipid parameters, biomarkers of atherosclerosis, the degree of arteriosclerosis, blood glucose parameters, blood pressure, anthropometric parameters, health-related quality of life, and the changes in the general symptoms of cold and hot patterns will be measured as secondary outcomes. Adverse events and laboratory test results will be investigated to assess the safety. Changes in the gut microbiome before and after intervention will also be assessed as an exploratory outcome through next-generation sequencing. Data will be recorded in electronic case report forms and analyzed using SAS® Version 9.4.

**Discussion:**

This is a rigorously designed pilot clinical trial to explore the effect and safety of Hwangryunhaedok-tang compared to placebo control for patients with hyperlipidemia, thereby potentially facilitating better management of hyperlipidemia. The results of this pilot study could form the foundation for a future large-scale, confirmatory clinical trial.

**Trial registration:**

Clinical Research Information Service KCT0004564. Registered on December 18, 2019

## Background

Hyperlipidemia refers to high blood levels of total cholesterol (TC), low-density lipoprotein cholesterol (LDL-C), or triglyceride (TG) [1]. Recently, the prevalence of hyperlipidemia has increased due to aging, diet, and lifestyle changes. In the USA, it is reported that 38.2% of adults have elevated TC levels [2]. In particular, hypercholesterolemia showed a significant increase, and the prevalence of hypercholesterolemia in the Republic of Korea more than doubled over 10 years from 10.7% in 2007 to 21.5% in 2017 according to the National Health and Nutrition Survey data in the Republic of Korea [3, 4]. In addition, attention has been focused on the correlation between hyperlipidemia and coronary artery disease. One of the reasons for the increase in coronary artery disease in the Republic of Korea despite the rapid decrease in cerebrovascular diseases is estimated to be an increase in hyperlipidemia [4]. It is also estimated that hypercholesterolemia is responsible for 56% of ischemic heart diseases and 18% of strokes worldwide [5].

Since hyperlipidemia usually has no symptoms, a screening test is essential and primary treatments include lifestyle changes such as diet control, exercise, and smoking cessation [4]. The use of conventional medication is based on a comprehensive assessment of cardiovascular disease risk and LDL-C levels [4]. In particular, conventional medication should be considered when LDL-C levels are still high after several weeks or months of lifestyle changes in patients without cardiovascular diseases [4]. Currently, statins are used as the first choice medications for treating hyperlipidemia; however, adverse reactions such as dyspepsia, heartburn, abdominal pain, liver toxicity, myalgia, rhabdomyolysis, and diabetes have emerged as problems [6, 7]. Therefore, the demand for treatment using botanic materials that can control blood lipid levels from the early stage of hyperlipidemia and can be safely taken for a long time is increasing.

Hyperlipidemia is regarded as a category of phlegm-dampness and static blood in East Asian traditional medicine (EATM) theory and treatment methods such as “dispel phlegm and eliminate dampness,” “activate blood and resolve stasis,” “tonify and replenish the middle qi,” and “clear heat and purge fire” have been proposed [8, 9]. Hwangryunhaedok-tang (HHT; Huang-lian-jie-du-tang in Chinese, Oren-gedoku-to in Japanese) is an herbal prescription comprised of four herbs that clear heat, dry dampness, purge fire, and detoxify and therefore, according to the EATM theory, HHT can treat hyperlipidemia. Additionally, it is widely used for cardiovascular and cerebrovascular diseases, such as hypertension, hyperlipidemia, and stroke in EATM [10, 11]. It has been reported that HHT showed LDL-C and TC lowering effects by inhibiting 3-hydroxy-3-methyl-glutaryl-coenzyme A (HMG-CoA) reductase in animal models of hyperlipidemia [12]. In addition, in the high-cholesterol animal model, the administration of HHT for 8 weeks significantly prevented the progression of thoracic aortic plaque with an anti-oxidative effect [13]. In particular, *Berberine*, a component of HHT, has been reported to reduce TC and LDL-C levels and increase high-density lipoprotein cholesterol (HDL-C) levels in animals and humans through mechanisms such as absorption of cholesterol in the intestine, removal of LDL receptor-mediated LDL-C, and conversion of cholesterol into bile [14]. In a randomized controlled trial (RCT) comparing HHT and statins in patients with hyperlipidemia, both groups exhibited significantly decreased TC, TG, and LDL-C and significantly increased HDL-C after treatment, compared to the levels before treatment. However, there was no significant difference between the two groups showing results in favor of HHT after treatment, with the exception of the fasting blood glucose level [15]. Furthermore, when HHT plus statin and statin alone were compared in patients with both hypertension and hyperlipidemia, inflammatory factors were significantly improved in the HHT plus statin group [16].

Although there is experimental and clinical evidence of the application of HHT for the treatment of hyperlipidemia as described above, and HHT has been used frequently in clinical settings of EATM, no studies have been conducted to evaluate its effect on blood lipid metabolism compared to placebo in patients with hyperlipidemia. Therefore, the aim of this pilot study is to explore the feasibility and preliminary effectiveness and safety of HHT for patients with hyperlipidemia.

## Methods/design

### Study design

**Fig. 1:**
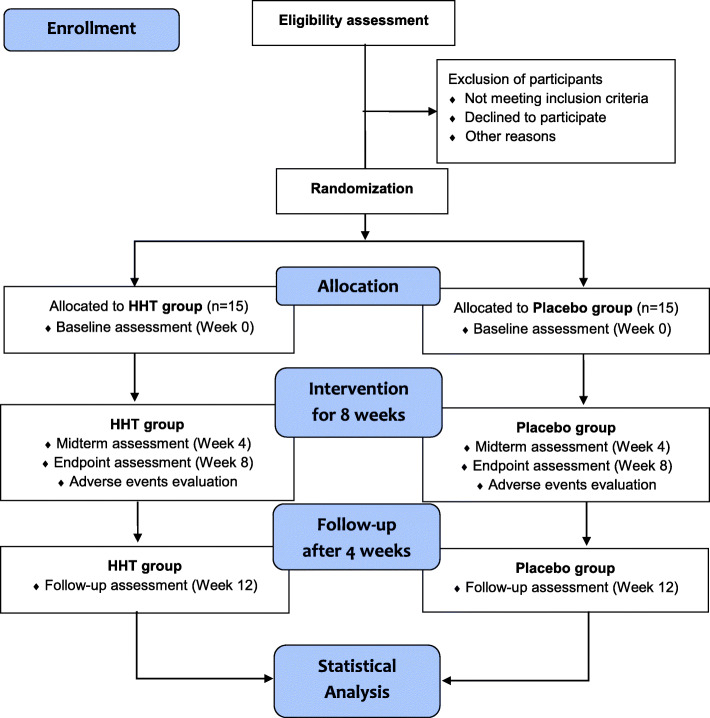
Flow diagram of the trial

This is a study protocol for a randomized, double-blind, placebo-controlled, parallel, investigator-initiated, pilot clinical trial. The study period will include 8 weeks of medication and 4 weeks of follow-up. The study procedure is summarized in the Consolidated Standards of Reporting Trials (CONSORT) diagram (Fig. 1), and the schedule of enrolment, intervention, and assessments is summarized in Table 1. The trial will be performed according to the Declaration of Helsinki and Good Clinical Practice Guidelines. In addition, the study protocol follows the CONSORT Extension for Chinese Herbal Medicine Formulas 2017 [17] and Standard Protocol Items: Recommendations for Interventional Trials 2013 statement (Additional file 1) [18].

**Table 1:**
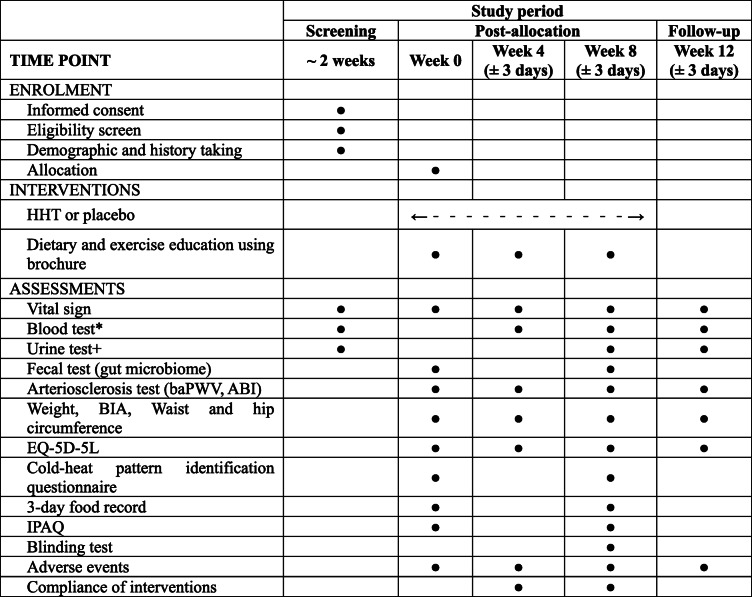
Schedule of enrolment, interventions, and assessments

*Abbreviations*: *ABI* ankle-brachial index, *baPWV* brachial-ankle pulse wave velocity, *BIA* bioelectrical impedance, *EQ-5D-5L* five-level EuroQol-5 dimensions, *HHT* Hwangryunhaedok-tang, *IPAQ* international physical activity questionnaire

*Including blood lipid parameters, biomarkers of atherosclerosis, blood glucose parameters, hematological test, thyroid hormone test, and liver and renal function test, depends on the time point

^+^Including human chorionic gonadotropin urine test only for women in their childbearing years at the screening visit

### Recruitment

The study will be performed at the Daejeon Korean Medicine Hospital of Daejeon University. The trial will be advertised through offline and online recruitment notices, including posters and webpages. Investigators will provide research information such as objectives, procedures, and potential benefits and risks to participants through standardized interviews prior to their participation and informed consent will be obtained from all participants. Participants will be allowed to withdraw from the study at any time without disadvantage and will be immediately notified when new information regarding the study is obtained.

### Inclusion criteria

1. Adults aged 19 years or over but under 65 years at the screening visit
2. 130 mg/dl ≤ baseline LDL-C levels ≤ 250 mg/dl
3. Those who have voluntarily signed written informed consent forms approved by the institutional review board (IRB) after sufficient explanation about this study

### Exclusion criteria

1. Having a history of unstable angina, myocardial infarction, transient ischemic attack, cerebrovascular disease, coronary artery bypass surgery, coronary intervention, or abdominal aneurysm within 3 months prior to screening
2. Use of statins, ezetimibe, fibric acid derivatives, proprotein convertase subtilisin/kexin type 9 (PCSK9) inhibitors, cholestyramine, nicotinic acid, omega-3 fatty acids, systemic steroids, diuretics, amiodarone, and cyclosporin, which can affect the lipid profile within 4 weeks prior to screening (however, the use of 4 g omega-3 fatty acids or less per day and short-term use of 6 mg or less per day of dexamethasone or equivalent corticosteroids within 5 days, which would not affect lipid levels, are allowed [19, 20])
3. Uncontrolled hypertension with systolic blood pressure of more than 180 mmHg or diastolic blood pressure of more than 110 mmHg
4. Uncontrolled diabetes mellitus with HbA1c of more than 9% or fasting blood glucose of more than 160 mg/dl
5. Uncontrolled thyroid function (thyroid-stimulating hormone (TSH) levels ≥ 1.5 times the upper limit of normal)
6. TG levels of more than 500 mg/dl
7. Severe liver or renal disease (aspartate aminotransferase (AST) or alanine aminotransferase (ALT) levels ≥ 3 times the upper limit of normal or creatinine levels ≥ 2 times the upper limit of normal)
8. Participants with a history of alcohol abuse or drug abuse within the past year
9. Women who are pregnant or lactating, or women who do not agree to use effective methods of contraception during the clinical trial
10. Participants with genetic problems, such as galactose intolerance, Lapp lactase deficiency, or glucose-galactose malabsorption
11. Participants with known hypersensitivity to the investigational products
12. Participants who had taken other investigational products within 3 months
13. Participants who are judged by investigators to be unsuitable for participation in the clinical trial for other reasons

### Randomization and allocation concealment

An independent statistician (OJK) will generate a random assignment code using the block randomization method in block sizes of 2 and 4 with no stratification in SAS® Version 9.4 (SAS Institute Inc., Cary, NC). Participants that satisfy the eligible criteria will be assigned to the HHT or placebo granule group at the baseline visit (Week 0), with a 1:1 allocation ratio. The generated code will be sealed in opaque envelopes and stored in double-locked cabinets. We will maintain allocation concealment throughout the study period by giving participants identically packaged, consecutively numbered drug containers.

### Blinding

The participants, investigators, outcome assessors, pharmacists, study monitors and data managers will be blinded to which medication will be administered to participants by using placebo granules as a control. The independent statistician who generated the random assignment code will provide the random assignment table directly to Hanpoong Pharm & Foods Co., Ltd. (Jeonju, Republic of Korea), and the company will pack and deliver investigational products to the hospital. The placebo granules will have the same formulation and properties as the HHT granules to prevent any bias in efficacy and safety assessments. Blinding will be maintained until all participants have completed the study, unless serious medical emergencies occur to participants. When unblinding is necessary such as in serious medical emergencies, a separate emergency code stored in double-locked cabinets will be opened by investigators. The investigators must document the reason for unblinding. To check whether the participant blinding is maintained during the study period, investigators will perform a blinding test on participants after 8 weeks of investigational product administration (Visit 4). Investigators will ask the participants which type of drug they think was administrated: “real drug,” “false drug,” or “do not know.” The success of blinding will be evaluated using the new blinding index and 95% confidence interval [21].

### Interventions

Eligible participants will be randomly assigned to receive HHT or placebo granules for 8 weeks. The HHT granule is made of an herbal extract, lactose hydrate, and corn starch. The herbal extract is composed of four herbs: *Coptidis Rhizoma*, *Phellodendri Cortex*, *Scutellariae Radix*, and *Gardeniae Fructus*. The raw materials (*Coptidis Rhizoma* 0.67 g, *Phellodendri Cortex* 1.0 g, *Scutellariae Radix* 1.0 g, *Gardeniae Fructus* 1.0 g) are extracted with 8 to 10 times the volume of water at 90–100 °C for 3–4 h. The extract is filtered and the filtrate is concentrated under reduced pressure at 60 °C or lower to obtain the herbal extract. The placebo granule is made of lactose hydrate, corn starch, hydroxypropyl methylcellulose, ginseng-flavored powder, and caramel coloring.

The HHT and placebo granules will be provided by Hanpoong Pharm & Foods Co., Ltd. (Jeonju, Republic of Korea) and are manufactured so that there is no difference between them in shape, color, smell, or taste. A blinded pharmacist will check the storage, dispensation, and quality of the investigational product.

Participants will be instructed to take one pack (3.0 g) at a time, three times per day for 8 weeks. The dosage regimen is based on authorization by the Korea Ministry of Food and Drug Safety. Participants will be instructed to return unopened packages of the investigational product to check their adherence to treatment. If any adverse events occur, investigators will take appropriate action depending on the severity of the adverse event and the causal relationship with the investigational product.

Co-administration of medications that are being used for the management of metabolic diseases, such as hypertension and diabetes, and dietary supplements that do not directly affect hyperlipidemia can be permitted during the study period. However, participants will be instructed not to change the dose or type of concomitant medications as much as possible and if they are changed, the investigators will record the details in the case report form (CRF).

Drugs that can cause dramatic changes in the gut microbiome (such as antibiotics and lactic acid bacteria) should be used cautiously only if needed, to minimize the effect on the gut microorganism, which is an exploratory outcome measure of this study. For participants who are already taking lactic acid bacteria, the investigators will instruct them not to change the dose or type as much as possible during the trial. If there is any change, the investigators will record the details in the CRF.

The use of statins, ezetimibe, fibric acid derivatives, PCSK9 inhibitors, cholestyramine, nicotinic acid, omega-3 fatty acids, systemic steroids, diuretics, amiodarone, and cyclosporine, which can affect the lipid profile, and the use of drugs that can affect thyroid function such as thyroxine will be forbidden during the study period. However, the use of 4 g omega-3 fatty acids or less per day and short-term use of 6 mg or less per day of dexamethasone or equivalent corticosteroids within 5 days, which would not affect lipid levels [19, 20], are allowed.

Dietary content and the physical activity level of participants are factors that may affect the results of this trial. In addition, gut microflora is affected by recent dietary intake. Therefore, we will compare food intake and the degree of physical activity before and after 8-weeks of investigational product administration by using a 3-day food record and the validated Korean version of international physical activity questionnaire short form [22], respectively. Participants in both groups will be educated and instructed to follow dietary and exercise guidelines for hyperlipidemia using a brochure based on the Korean Guidelines for the Management of Dyslipidemia [4].

### Early termination and dropout criteria

The criteria for early termination and dropout of individual participants are when the participants withdraw consent, request a discontinuation of the clinical trial, or violate the test plan. Based on the investigator’s judgment, participants will be removed from the study if they are no longer able to participate in the trial due to adverse reactions or concomitant disease or if they take prohibited drugs that can affect the outcome of the study. In addition, if it is judged that the efficacy evaluation is not suitable because the compliance rate with the investigational drug is less than 70%, or if the participants cannot be tracked, treatment will be discontinued early. If moderate or severe adverse reactions judged to be related to the clinical trial occur in more than 25% of the total participants, or if the required number of participants has not been enrolled despite a sufficient recruitment period, this clinical trial may be terminated early.

### Data collection

#### Background information

Investigators will collect the demographic and clinical information of participants and perform vital sign measurement and laboratory tests only for those participants who voluntarily sign the informed consent form at the screening visit. Demographic data include age, gender, height, weight, smoking history, and alcohol consumption. General clinical information includes medical history, treatment history, concomitant disease, and medication. The personal information of the participants will be de-identified by using a coded ID number and will be stored securely at the institution to protect confidentiality and ensure access is limited to the investigators involved in the study.

### Efficacy outcomes

The primary outcome is the blood level of LDL-C, which will be measured at baseline and 4, 8, and 12 weeks after randomization. In this study, the primary endpoint is 8 weeks post-treatment. The secondary outcomes are other blood lipid parameters including TC, TG, HDL-C, non-HDL-C, non-HDL-C/HDL-C, apolipoprotein (Apo) A1, Apo B, Apo B/Apo A1, lipoprotein (a), and atherogenic index of plasma (Log(TG/HDL-C), and atherosclerosis biomarkers including high-sensitivity C-reactive protein (hs-CRP), fibrinogen, and homocysteine, which will be measured at baseline and 4, 8, and 12 weeks after randomization. Using VP-1000plus (OMRON Healthcare Co., Kyoto, Japan), the degree of vessel hardening will be assessed through brachial-ankle pulse wave velocity (baPWV). The degree of narrowing due to vascular lesions will be assessed by measuring the blood pressure difference between the ankle and arm using the ankle-brachial index (ABI) to determine the degree of arteriosclerosis. Blood glucose parameters including fasting glucose and HbA1c, blood pressure, and anthropometric parameters including body weight, body fat percentage, body fat mass, skeletal muscle mass, waist circumference, hip circumference, and waist-to-hip ratio will also be measured. The health-related quality of life before and after taking the investigational product will be measured using the five-level EuroQol-5 dimensions (EQ-5D-5L). The EQ-5D-5L is composed of 25 items in five domains including mobility, self-care, usual activities, pain/discomfort, and anxiety/depression. Participants will respond to each item with five response levels: no problems (level 1), slight, moderate, severe, and extreme problems (level 5) [23]. The Korean version of EQ-5D–5 L will be used after permission from the EuroQol Research Foundation. Blood lipid parameters, atherosclerosis biomarkers, the degree of arteriosclerosis, blood glucose parameters, blood pressure, anthropometric parameters, and EQ-5D-5L will be measured at baseline and 4, 8, and 12 weeks after randomization.

The changes in the cold and hot patterns for participants will be measured using a cold-heat pattern identification questionnaire at baseline and 8 weeks post-treatment. The cold-heat pattern identification questionnaire was developed by the Korea Institute of Oriental Medicine (KIOM) and is a self-report questionnaire consisting of eight items related to cold patterns and seven items related to hot patterns [24]. The reliability and validity of the questionnaire have been confirmed [25]. As an exploratory outcome measure, fecal samples of participants before and after taking the investigational product (baseline and 8 weeks post-treatment) will be examined to identify changes in the gut microbiome through next-generation sequencing. The time points for each outcome measure are presented in detail in Table 1.

### Safety outcomes

The safety of the investigational product will be measured by comparing the laboratory liver and renal function test results (AST, ALT, blood urea nitrogen, and creatinine) before and after intervention in each group. Investigators will also examine the adverse events through medical examinations and participant reports at each visit, and all adverse events during the study will be systematically recorded in the CRF regardless of the relationship with the intervention provided in this study. Investigators will ensure that all participants with adverse reactions receive adequate medical attention and follow-up until the symptoms are resolved.

### Sample size

This is the first study to explore the feasibility and lipid-improving effect and safety of HHT granules compared with placebo granules. Therefore, a formal sample size calculation is not required [26]. Based on a study recommending a minimum of 12 participants per group for a pilot study [27], we selected 15 participants per group as a sample size for this trial, considering a 20% dropout rate.

### Statistical analysis

An independent statistician will conduct statistical analysis using SAS® Version 9.4 (SAS Institute Inc., Cary, NC). In principle, efficacy analysis will be conducted using a full analysis set according to the intention-to-treatment principle, including all randomly assigned participants receiving at least one evaluation after the investigational product has been prescribed at least once. If necessary, per protocol set (PPS) analysis will also be conducted including only participants who have completed the entire process as described in the protocol and have no significant violations. Participants less than 70% compliant with the investigational product will be excluded from the PPS analysis. Safety analysis will include all data obtained from participants who have taken at least one investigational product.

The demographic and general clinical characteristics of participants will be summarized by group. Continuous data will be presented as mean and 95% confidence intervals or median according to the distribution of the data and dichotomous data as frequency and percentage. Continuous data will be analyzed using the independent *t* test or Wilcoxon rank sum test, and dichotomous data will be analyzed using the chi-square test or Fisher’s exact test.

The primary outcome in this study is the mean change in LDL-C from baseline to week 8. A two-sided test with a significance level of 5% will be performed using an analysis of covariance with baseline as the covariate and the treatment group as the fixed factors. The intra-group changes of outcome measures from baseline to post-treatment will be analyzed using paired *t* test or Wilcoxon signed rank test for continuous variables and the chi-square test or Fisher’s exact test for dichotomous variables. Furthermore, repeated measures analysis of variance will be used to compare the differences in trends between visits in each group. Missing values will be replaced with the multiple imputation method. If necessary, a subgroup analysis can be performed by categorizing the participant’s initial characteristics at the screening or baseline visit.

### Data monitoring

We will use a web-based electronic CRF developed by Medidata (NY, USA) for data collection and verification. It will be managed by data management team from KIOM. The electronic CRF is accessible only to investigators who are directly involved in the clinical trial and received the relevant education. To ensure the data quality, the range of data values will be set in advance. Although data entry will be performed only once by the investigators, data quality will be managed with two verification processes for each clinical research associate and data manager. A clinical research associate at KIOM (ARK), a supporting institution, will visit the hospital regularly to monitor protocol violations, recruitment rate, document reporting, and adverse events (including serious adverse events) during the study period. The detected item will be properly resolved through discussions with investigators. No formal data monitoring committee will be convened. There is currently no planned auditing. However, auditing can be conducted to preserve the integrity of the trial by the quality assurance team at the supporting institution, independent from the investigators.

### Patient and public involvement

This study is designed to explore the feasibility and preliminary effectiveness and safety of HHT for patients with hyperlipidemia. HHT is an herbal drug approved as a generic drug by the Korea Ministry of Food and Drug Safety in the Republic of Korea and is easily accepted by patients. Using HHT for hyperlipidemia may improve blood lipid levels, preventing cardiovascular disease and improving the quality of life. In addition, the outcome measures used in this trial are considered important endpoints for patients with hyperlipidemia in clinical practice. However, patients were not involved directly in developing the study design and will not be involved in the recruitment or conduct of the study. In addition, there is no plan to communicate the results of this study to trial participants. If the participants request the results of the study after the clinical trial has been completed, we will provide a summary of the results.

### Ethics and dissemination

The trial protocol has been approved by the Korea Ministry of Food and Drug Safety (approval number: 32578) and the IRB of Daejeon Korean Medicine Hospital of Daejeon University (approval number: DJDSKH-19-DR-18). The study was registered at the Clinical Research Information Service (CRIS) (registration number: KCT0004564). Any modifications to the protocol will be reapproved by the IRB prior to implementation and documented in CRIS. All participants will receive a detailed description of the research process from a licensed Korean medicine doctor and be asked to sign an informed consent form prior to participation. In addition, consent forms for the use of human biological materials will be acquired for the handing of fecal samples from the participants. The results of this study will be submitted for publication in peer-reviewed journals and can be disseminated through conference presentations.

## Discussion

This is a study protocol for a randomized, double-blind, placebo-controlled, parallel, investigator-initiated, pilot clinical trial evaluating the effect of HHT on patients with hyperlipidemia. We will evaluate the preliminary efficacy and safety of 8-week administration of HHT compared with placebo granules and assess the feasibility of large-scale RCTs. According to the 2019 European Society of Cardiology and European Atherosclerosis Society Guidelines for the management of dyslipidemias, LDL-C analysis is recommended as the primary lipid analysis method for screening, diagnosis, and management [28]. Therefore, we set the inclusion criteria and primary outcome measure for our study as LDL-C, not TC or TG. In addition, we set 250 mg/dl as the upper bound for LDL-C in the inclusion criteria by referencing the guidelines for clinical trials for hyperlipidemia treatment by the Korean Ministry of Food and Drug Safety [29], considering that this study will include participants who are not taking conventional lipid-lowering drugs. According to a consensus document on clinical research in Chinese medicine, it is recommended to recruit participants according to the conventional medicinal diagnosis, and then to perform pattern identification before randomization. Recruiting participants based on pattern identification can be considered if a limited number of identifiable patterns exist [30]. To the best of our knowledge, there has been no consensus on standardized pattern identification for hyperlipidemia. Therefore, we will recruit hyperlipidemia participants according to the conventional medical diagnosis, and then perform cold-heat pattern identification before randomization.

Although there is a previously published clinical trial evaluating the efficacy and safety of HHT in hyperlipidemia, it directly compared HHT and Simvastatin, and the duration of treatment was 4 weeks [15]. Our study attempts to evaluate the efficacy of HHT compared to placebo in patients with hyperlipidemia who are not taking conventional lipid-lowering drugs such as statins, and the treatment period was set to 8 weeks. Therefore, there was no reference to calculate the formal sample size, and if this pilot study design is feasible, a confirmatory clinical trial will be conducted by calculating the appropriate sample size based on the results of this study.

Herbal medicine contains multiple active components that act on multiple targets and has the potential to overcome the limitations of conventional medications with a single active constituent [31]. According to an RCT comparing HHT with statin in patients with hyperlipidemia, there was no statistically significant difference in blood lipid parameters between the two groups, but fasting blood glucose was significantly lower in the HHT group than in the statin group [15]. In addition, comparison of the administration of HHT plus statin and statin alone to patients with both hypertension and hyperlipidemia showed a significant improvement in inflammatory factors in favor of HHT plus statin [16]. Furthermore, when HHT was administered to obese patients, body weight, body fat, and waist circumference decreased significantly [32]. The results of these studies suggest the efficacy of HHT as a single treatment for metabolic syndrome, which is a complex lifestyle disease including hyperlipidemia, glucose metabolism abnormalities, hypertension, and obesity. Therefore, we set blood glucose parameters, blood pressure, and anthropometric parameters as the secondary outcome measures.

Although hyperlipidemia itself is asymptomatic, it is the most important risk factor for atherosclerosis, the major cause of cardiovascular disease [33]. Studies have suggested that non-invasive measurements of baPWV and ABI might be used as markers of atherosclerosis [34, 35]. In addition, hs-CRP, fibrinogen, and homocysteine have been suggested as biomarkers of atherosclerosis [36–38]. Therefore, we will measure baPWV, ABI, hs-CRP, fibrinogen, and homocysteine as markers of atherosclerosis to investigate the effect of HHT on atherosclerosis.

In EATMs, HHT is a representative heat-clearing medicine and its effect on alleviating fever in animal models has been demonstrated [39]. Therefore, we will examine the changes in cold and hot patterns in participants using a validated cold-heat pattern identification questionnaire after 8-weeks of HHT administration.

Recent studies have shown a close relationship between hyperlipidemia and gut microorganisms and patients with hyperlipidemia often exhibit intestinal microbiota disorders that might further impair lipid metabolism [40]. Recent research showed that *Berberine*, the main component of *Coptidis Rhizoma,* improves lipid metabolism in the liver by changing gut microbiota and by regulating bile acid metabolism and the farnesoid X receptor pathway in the intestine [41]. Therefore, we will investigate the changes in the gut microbiome before and after HHT administration and explore the effects of HHT on the composition, diversity, and species abundance of gut microorganisms in patients with hyperlipidemia.

Side effects from the use of HHT may include gastrointestinal reactions such as anorexia, stomach discomfort, nausea, and vomiting, and hypersensitivity reactions such as rash and urticaria. In rare cases, interstitial pneumonia or liver dysfunction may occur. However, according to related clinical trials in which HHT was administered to patients with hyperlipidemia, no serious adverse reactions were observed and no abnormalities were found in blood and urine tests and electrocardiograms [15, 42].

In our study, there is a limitation due to the small sample size of 15 participants per group and this may not be enough to draw a definite conclusion of the efficacy of intervention. However, to the best of our knowledge, this is the first RCT assessing the effect of HHT on patients with hyperlipidemia. If the treatment effect and safety of HHT compared with placebo granules is found to be promising in this study and the study design is feasible, we will design a confirmatory, large-scale RCT based on the results of the study to draw definitive conclusions regarding the efficacy of HHT.

In conclusion, this article presents the protocol for a rigorously well-designed, double-blind, placebo-controlled, pilot RCT on HHT for patients with hyperlipidemia. Our study has the strengths of using placebo granules as the control and using the objective blood lipid parameter, LDL-C, as the primary outcome, to minimize performance bias and detection bias. The findings of this study are expected to provide a base for large-scale, confirmatory RCT to confirm the efficacy and safety of HHT for the treatment of patients with hyperlipidemia. Additionally, stronger evidence of HHT as a treatment option for hyperlipidemia may be provided to clinicians, patients, and researchers.

### Trial status

This trial has not started participant recruitment yet. Protocol version number and date: version 1.2 (December 6, 2019). Date recruitment will begin: January 15, 2020. Approximate date when recruitment will be completed: December 31, 2020.

## Supplementary information

**Additional file 1.** SPIRIT 2013 Checklist: Recommended items to address in a clinical trial protocol and related documents.

**Additional file 2.** Protocol versions.

## Data Availability

The datasets used or analyzed (or both) during the current study are available from the corresponding author on reasonable request.

## Abbreviations

ABI: Ankle-brachial index
ALT: Alanine aminotransferase
Apo: Apolipoprotein
AST: Aspartate aminotransferase
baPWV: Brachial-ankle pulse wave velocity
CONSORT: Consolidated Standards of Reporting Trials
CRF: Case report form
CRIS: Clinical Research Information Service
EATM: East Asian traditional medicine
EQ-5D-5L: Five-level EuroQol-5 dimensions
HDL-C: High-density lipoprotein cholesterol
HHT: Hwangryunhaedok-tang
HMG-CoA: 3-Hydroxy-3-methyl-glutaryl-coenzyme A
hs-CRP: High-sensitivity C-reactive protein
IRB: Institutional review board
KIOM: Korea Institute of Oriental Medicine
LDL-C: Low-density lipoprotein cholesterol
PCSK9: Proprotein convertase subtilisin/kexin type 9
PPS: Per protocol set
RCT: Randomized controlled trial
TC: Total cholesterol
TG: Triglyceride
TSH: Thyroid-stimulating hormone
