# Supplementary material for: Efficacy of Hwangryunhaedok-tang (Huang-lian-jie-du-tang, Oren-gedoku-to) for patients with hyperlipidemia: a study protocol for a randomized, double-blind, placebo-controlled, parallel, investigator-initiated clinical trial
Source: Trials. 2020 Aug 27;21:750. doi: 10.1186/s13063-020-04695-3 (PMC7450942; doi:10.1186/s13063-020-04695-3)
Supplement: Supplementary file 2 — Additional file 2. Protocol versions. [file 13063_2020_4695_MOESM2_ESM.docx]

**Additional file 2**. Protocol versions

| **Version** | **Date** | **Action** |
| --- | --- | --- |
| 1.0 | 2019-Sep-18 | Protocol draft development |
| 1.1 | 2019-Nov-1 | Amendment of Investigational New Drug review |
| 1.2 | 2019-Dec-6 | Revision following institutional review board’s comments |
| 1.3 | 2020-Jan-17 | Additional minor amendment by researchers |
